# Update on the core and developing cerebrospinal fluid biomarkers for Alzheimer disease

**DOI:** 10.3325/cmj.2014.55.347

**Published:** 2014-08

**Authors:** Mirjana Babić, Dubravka Švob Štrac, Dorotea Mück-Šeler, Nela Pivac, Gabrijela Stanić, Patrick R. Hof, Goran Šimić

**Affiliations:** 1 Department of Neuroscience, Croatian Institute for Brain Research, University of Zagreb School of Medicine, Zagreb, Croatia; 2 Division of Molecular Medicine, Ruđer Bošković Institute, Zagreb, Croatia; 3 Department of Pathology and Cytology, “Sveti Duh” Clinical Hospital, Zagreb, Croatia; 4 Fishberg Department of Neuroscience and Friedman Brain Institute, Icahn School of Medicine at Mount Sinai, New York, NY, USA

## Abstract

Alzheimer disease (AD) is a complex neurodegenerative disorder, whose prevalence will dramatically rise by 2050. Despite numerous clinical trials investigating this disease, there is still no effective treatment. Many trials showed negative or inconclusive results, possibly because they recruited only patients with severe disease, who had not undergone disease-modifying therapies in preclinical stages of AD before severe degeneration occurred. Detection of AD in asymptomatic at risk individuals (and a few presymptomatic individuals who carry an autosomal dominant monogenic AD mutation) remains impractical in many of clinical situations and is possible only with reliable biomarkers. In addition to early diagnosis of AD, biomarkers should serve for monitoring disease progression and response to therapy. To date, the most promising biomarkers are cerebrospinal fluid (CSF) and neuroimaging biomarkers. Core CSF biomarkers (amyloid β_1-42_, total tau, and phosphorylated tau) showed a high diagnostic accuracy but were still unreliable for preclinical detection of AD. Hence, there is an urgent need for detection and validation of novel CSF biomarkers that would enable early diagnosis of AD in asymptomatic individuals. This article reviews recent research advances on biomarkers for AD, focusing mainly on the CSF biomarkers. In addition to core CSF biomarkers, the potential usefulness of novel CSF biomarkers is discussed.

According to the World Alzheimer Report, in 2009 35.6 million people worldwide suffered from dementia. Alzheimer disease (AD) is the major primary cause of dementia and affects 60%-80% of demented people (1). Because of the longer life span and increasing number of elderly people, it is estimated that by 2050 this number will reach 115.4 million (1). In absence of a cure for AD, current medications only alleviate the symptoms and have generally been tested principally only in late-stage AD patients. The pathological process in AD brain starts at least 10-20 years before the occurrence of the first dementia symptoms (2). Therefore, it is crucial to treat asymptomatic individuals, in whom degeneration is not yet severe, with disease-modifying drugs (Figure 1) (3). Reliable biomarkers are essential as they are necessary for early AD detection at preclinical stages. Besides an important role in diagnostics, biomarkers can also provide insight into the AD pathogenesis.

**Figure 1:**
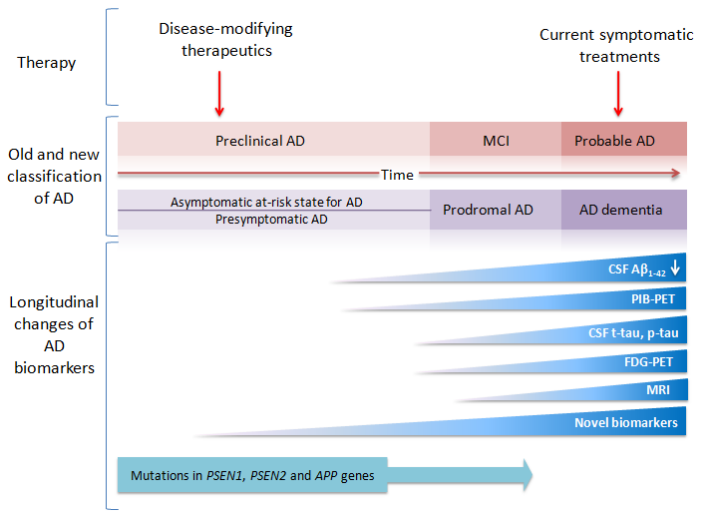
Longitudinal changes of Alzheimer disease biomarkers during the disease progression.

Biomarkers are usually analyzed in bodily fluids such as blood, urine, or the cerebrospinal fluid (CSF), but data collected with brain imaging methods are also considered as biomarkers (4-6). To be accepted as such, a new marker of AD must fulfill two conditions: it must be evaluated in at least two independent peer-reviewed cross-sectional clinical studies and be confirmed neuropathologically at autopsy (7). The key features of an ideal AD biomarker are sensitivity (probability of AD detection) and specificity (differentiation of AD patients from healthy individuals and patients with other primary causes of dementia) above 85%. Additional important characteristics are availability, non-invasiveness, reasonable cost, and potential for repeated measurements (Table 1) (7,8). A strong biomarker also should have high early diagnostic sensitivity and pathological specificity, and correlate with disease progression (Table 2). In general, biomarkers are divided into two groups: biomarkers of exposure and biomarkers of disease. Biomarkers of exposure serve for the estimation of disease risk factors, while biomarkers of disease are used in screening (prognostic markers), diagnostic tests for early disease detection (diagnostic markers), and monitoring disease progression (staging markers). This group of biomarkers is also used in monitoring response to therapy (9,10). As such, reliable biomarkers are extremely important for the selection of patients for clinical trials, and consequently for treatment validation (11).

**Table 1:** Characteristics of Alzheimer disease (AD) biomarkers

	Advantages	Disadvantages
**Cerebrospinal fluid biomarkers**	High sensitivity and specificity; best reflection of pathological processes in the AD brain; diagnostic utility confirmed by many studies	Invasive sample collection by lumbar puncture; expensive ELISA tests; inter- and intra-laboratory variability; follow-up of the patients mostly not possible due to lumbar puncture
**Neuroimaging biomarkers**	High sensitivity and specificity; noninvasive; diagnostic utility confirmed by many studies	Sophisticated techniques; expensive radiotracers; not widely distributed
**Plasma biomarkers**	Minimally invasive; possible follow-up of patients; screening of healthy population	Low sensitivity and specificity; conflicting results; still unavailable suitable plasma biomarkers
**Genetic biomarkers**	Excellent for prediction of familial AD; noninvasive; low-cost genetic tests; screening of healthy population	No available genetic biomarkers for sporadic AD

**Table 2:** Diagnostic usefulness of established Alzheimer disease biomarkers

Biomarkers	Pathological specificity	Early diagnostic sensitivity	Correlation with disease progression
Neuropsychological testing	+	++	++
Amyloid β_1-42_	++	+	+
t-tau	++	++	+
p-tau	+++	+++	++
Magnetic resonance imaging	+	++	+++
Positron emission tomography	+	++	+++

*Minimum (+), moderate (++), maximum (+++).

The existing diagnostic tests of AD are mostly based on neuropsychological assessment (12), which remains inadequate for early detection and differentiation of AD from other types of dementia, such as vascular dementia (VaD), frontotemporal dementia (FTD), or Lewy body disease (LBD). Probable AD is diagnosed after the onset of the first symptoms when degeneration is already substantial (13). Consequently, the European Federation of Neurological Societies recommended additional tests for AD diagnosis, including assessment of brain volumetry using magnetic resonance imaging (MRI) and measurement of tau protein in the CSF (14,15). Furthermore, Dubois et al (16) revised the generally accepted NINCDS-ADRDA (National Institute of Neurological and Communicative Disorders and Stroke and Alzheimer Disease and Related Disorders Association) criteria for AD diagnosis (17). In addition to the core diagnostic criteria, which mostly refer to monitoring of episodic memory impairment using a battery of neuropsychological tests, supportive criteria should be considered, which include abnormal CSF biomarkers, medial temporal lobe atrophy, reduced glucose metabolism in the temporal and parietal regions, or the presence of an ϵ4 allele in the gene for apolipoprotein E (ApoE) (16). This refines the diagnosis by defining the stages of AD progression: at-risk state for AD (asymptomatic individuals with positive biomarkers), presymptomatic AD (autosomal-dominant mutation carriers), prodromal AD (mild episodic memory loss, positive biomarkers), and AD dementia (severe episodic memory loss, positive biomarkers) (Figure 1) (16). Through the initiation and work of the Biomarker Consortium (18) and the Alzheimer Disease Neuroimaging Initiative (19), scientists and clinicians attempted to emphasize the importance of biomarkers in early AD detection in asymptomatic individuals.

We reviewed the existing biomarkers of AD, with a special focus on the CSF. Also, alterations in novel CSF biomarkers, especially CSF neurotransmitters, during AD, their role in the disease process, and potential diagnostic applications are discussed.

## CSF core biomarkers

### Amyloid β protein

Senile plaques, one of the major neuropathological hallmarks of AD, are formed as a result of excessive amyloid β (Aβ) protein production, aggregation, and deposition in the brain (20). In early-onset familial AD, these pathological processes are caused by mutations in the genes encoding proteins involved in the production of Aβ, whereas in sporadic AD an imbalance in formation and removal of Aβ is observed. While in AD, pathological Aβ_1-42_ is formed after processing of the amyloid precursor protein (APP) through the amyloidogenic pathway (β-secretase [BACE1] pathway), in healthy individuals prevails the non-amyloidogenic pathway (α-secretase pathway), in which Aβ_1-42_ is not formed (21).

Due to molecular exchange at the brain/CSF interface, pathological processes in the brain are frequently reflected in the CSF (22). Thus, numerous studies investigated the decrease in Aβ_1-42_ in the CSF of AD patients by enzyme-linked immunosorbent assay (ELISA) (23-26). A 50% lower Aβ_1-42_ diffusion in the CSF can be explained by its deposition in senile plaques (27). However, because a reduction of CSF Aβ_1-42_ was noticed in other diseases like Creutzfeldt-Jakob disease (CJD) and multiple system atrophy, in which senile plaques are not formed, it is believed that the reduction of Aβ_1-42_ levels in the CSF can be also mediated by other mechanisms (28,29). For instance, it can be a result of Aβ_1-42_ binding either to ApoE, cystatin C, or β-trace protein, accumulation in the form of oligomers, or sequestration in membranes (30).

CSF Aβ_1-42_ levels show high sensitivity (78%-100%) but insufficient specificity (47%-81%) in differentiating AD patients from healthy controls (31). Although the accuracy of established cut-off levels is still discussed, levels of Aβ_1-42_ lower than 500 pg/mL are generally considered compatible with AD (32). Levels of Aβ_1-42_ are not changed with aging and in pathological conditions like Parkinson disease, progressive supranuclear palsy, alcoholic dementia, depression, and stroke (29,33). However, Aβ_1-42_ decrease is also observed in LBD, FTD, VaD, amyotrophic lateral sclerosis, multiple system atrophy, CJD, and in neuroinflammation, resulting in insufficient Aβ_1-42_ specificity (28,29,33-35). Yet, Aβ_1-42_ reduction is observed very early, much before the occurrence of the first AD symptoms (36). Thus, measurement of Aβ_1-42_ in CSF can facilitate the diagnostics of incipient AD in patients with mild cognitive impairment (MCI; MCI-AD) (37). Recent studies have assessed the diagnostic usefulness of Aβ_1-42_, and showed that it is very unstable (degrading after 2 freeze-thaw cycles) and forms only 10% of the total amyloid proteins in CSF. A more precise AD biomarker than Aβ_1-42_ alone is the Aβ_1-42_ /Aβ_1-40_ ratio, even though Aβ_1-40_ is slightly increased or unchanged in the CSF of AD patients (38).

### Tau protein

Tau protein is the major component of neurofibrillary tangles (NFT), the other key neuropathological hallmark of AD (39). It is located principally in the axons, connects microtubules, and regulates axonal length, stability, and rigidity (40). Hyperphosphorylation of tau proteins leads to detachment of tau from microtubules, degradation of microtubules and consequently axons, and results in neuronal death (41). Abnormally phosphorylated tau proteins further accumulate and form NFT (42-44).

*Total tau in the CSF.* Axon degradation and neuronal death lead to the release of tau proteins in the CSF. Many studies confirmed an increase in total tau (t-tau) in the CSF of AD patients (25,26,45-47). Levels of t-tau vary from 300 to 900 pg/mL and can be increased by as much as 300% in comparison to healthy controls (48). Because CSF t-tau levels increase with aging, cut-off values are adjusted by age. For patients 51-70 years old, levels higher than 450 pg/mL are considered pathological, while above this age the cut-off value is 600 pg/mL (32). T-tau reached high sensitivity (84%) and specificity (91%) in differentiating AD patients from healthy individuals (48). These numbers should be taken with caution as elevation of CSF t-tau is also detected in VaD, FTD, CJD, stroke, and after traumatic brain injury (45-47,49-51). In all of these pathological conditions, t-tau is moderately elevated, except in CJD, where as a consequence of severe neuronal damage the levels of t-tau can reach 3000 pg/mL (52). Therefore, t-tau is not a reliable biomarker for differentiation of AD from other types of dementia. Its levels are normal in the CSF of geriatric patients with major depression (MD). As early AD symptoms coincide with MD symptoms, measurement of CSF t-tau enables correct differentiation between these two groups and adequate treatment of patients with MD (53). Using t-tau as a biomarker, it is possible to detect incipient AD in the group of MCI patients with high sensitivity and specificity (37). However, as in the case of Aβ_1-42_, it should be kept in mind that MCI can precede other types of dementia in which t-tau is also elevated (37).

*Phosphorylated tau in the CSF.* Out of the 85 (Dr Luc Buée, personal communication) possible phosphorylation sites (mainly on serines and threonines), tau protein is phosphorylated on approximately 71 sites in physiological or pathological conditions (54). Phosphorylation is regulated by numerous kinases, leading to different phosphorylation of tau at different stages of the disease (55). It has been reported that in AD there is an increase in phosphorylated tau (p-tau) to approximately 250% of control levels (24,45-48). Different p-tau epitopes have been measured in the CSF using ELISA method: threonine 231 (p-tau_231_), serine 199 (p-tau_199_), threonine 181 (p-tau_181_), serine 235, and serine 369/404 (31,47,56-58). As p-tau reflects pathology in AD brain better than t-tau, very high specificity (92%) and sensitivity (80%) was reported in differentiating AD patients from healthy controls (31). Unlike t-tau, a general indicator of degeneration and neuronal death, p-tau reflects the phosphorylation state of tau protein and the formation of NFTs in AD brain (59). However, it is still unknown what the main source of p-tau in the CSF is and whether neurons affected by tau pathology excrete p-tau in the extraneuronal space and by which mechanism. These gaps in knowledge question the assumption that p-tau accurately reflects NFTs in AD brain (60). Nevertheless, p-tau has more than 80% specificity in differentiating AD from other primary causes of dementia (61). Additionally, normal p-tau levels were found in pathological states like VaD, FTD, LBD, during depression, and after stroke, while fetal tau isoform is normally hyperphosphorylated during development (49,52,62,63). However, a moderate increase in p-tau was observed in CJD (52), as well as a decrease in both t-tau and p-tau in Parkinson disease (64). In a recent study, p-tau has been used as a biomarker for detection of MCI-AD patients (38).

The most studied p-tau biomarkers are p-tau_181_, p-tau_199_, and p-tau_231_. In most clinical studies, t-tau, p-tau_181_, and Aβ_1-42_ are measured in the CSF as a part of routine analyses, with a cut-off level for p-tau_181_ being 60 pg/mL (32,56,57). p-tau_181_ was confirmed as a good biomarker in differentiating AD from LBD and idiopathic normal pressure hydrocephalus (57,65). Additionally, Hansson et al (38) detected MCI-AD patients with 95% sensitivity and 83%-87% specificity by combining t-tau, Aβ_1-42_, and Aβ_1-42_/p-tau_181_ ratio. Regarding p-tau_199_ epitope, Itoh et al (66) detected AD using this biomarker with very high sensitivity and specificity (above 85%). Moreover, Boban et al (47) differentiated patients with FTD and AD with 88% accuracy by combining t-tau and p-tau_199_. However, p-tau_181_ and p-tau_231_ showed better results in early detection of AD (67). Elevation of p-tau_231_ levels and correlation with cognitive decline was reported in the group of MCI-AD patients (58,67). Additionally, a multicenter study by Hampel et al (61) showed that CSF p-tau_231_was a stable biomarker of MCI conversion to AD. p-tau_231_ was also considered as a potential biomarker for differentiation of AD from VaD, LBD, and FTD (23). In conclusion, if CSF concentrations of all three p-tau biomarkers are elevated, clinicians can be 90% confident that a patient is suffering from AD (68).

### Longitudinal changes of CSF biomarkers

Most studies on CSF biomarkers have had cross-sectional rather than longitudinal design. However, data from longitudinal studies could be very useful in monitoring the response to therapy. Recent studies have shown CSF biomarkers (Aβ_1-42_, t-tau, p-tau) to be stable from 6 months up to 2 years of disease progression and suitable for monitoring the CSF changes induced by therapy (69-71). On the other hand, Bouwman et al (72) reported an increase in Aβ_1-42_ and t-tau (but not p-tau) during AD progression, while two other studies indicated a decrease in Aβ_1-42_ in AD patients and in p-tau in the late stages of the disease (73,74). In addition, Toledo et al (75) described two distinct groups of participants with normal baseline CSF values: patients with stable and patients with abnormal longitudinal CSF biomarkers (decreasing Aβ_1-42_ and increasing p-tau_181_). They also reported that Aβ_1-42_ decrease precedes an increase in p-tau_181_, further supporting the notion that CSF Aβ_1-42_ changes appear before tau changes (75,76). The model of dynamic biomarkers proposed by Jack et al sheds light on these issues, confirming that CSF Aβ_1-42_ pathology precedes CSF tau pathology (Figure 1) (77,78). This model also suggests a sigmoid curve of abnormality of biomarkers during disease progression. According to this model, when dementia starts, most of the biomarkers have already reached the plateau phase (especially Aβ_1-42_) and do not change as much as in preclinical stages of disease (78). However, although Aβ_1-42_ CSF changes occur before tau changes, Braak et al confirmed the well-known finding that tau aggregation preceded plaque formation in AD brain (60).

## Novel CSF biomarkers

Besides core CSF biomarkers, other biomarkers could reflect AD pathological processes and improve the diagnostics of AD (25). These novel biomarkers are mostly related to Aβ metabolism, degeneration, inflammation, or lipid metabolism. The most useful novel biomarkers related to Aβ metabolism are CSF BACE1 activity, levels of APP isoforms (sAPPα and sAPPβ), Aβ oligomers, and C-terminal truncated Aβ isoforms (Aβ_1-37_, Aβ_1-38_, Aβ_1-39_, Aβ_1-14_, Aβ_1-15_, Aβ_1-16_) (79-83). In the CSF of AD patients there are also altered levels of neprilysin and cystatin C proteins involved in Aβ metabolism (84,85). Also, an increase in CSF neuromodulin (GAP43), neurofilament proteins, and visinin-like protein 1 (VILIP-1) reflects degeneration that occurs in the AD brain (86,87). Other potential biomarkers of degeneration are α-dystroglycan, precursor of neural cell adhesion molecule 1 (NCAM-120), neuronal pentraxin receptor (NPR), cocaine- and amphetamine-regulated transcript (CART), glial cell-derived neurotrophic factor (GDNF), and brain-derived neurotrophic factor (BDNF) (88-90). Disease progression results in the alteration of many inflammatory factors in the CSF, like interleukin 1 (IL-1), IL-6, tumor necrosis factor alpha (TNF-α), transforming growth factor beta (TGF-β), S100 calcium-binding protein A7 (S100A7), complement C1q, interferon–γ, and markers of microglial activation: chemokine (C-C motif) ligand 2 (CCL2), triggering receptor expressed on myeloid cells 2 (TREM2), and chitotriosidase (5,83,91-94). None of these proteins has been sufficient to make an AD diagnosis due to high variability among studies. However, chitinase-3-like protein 1 (YKL-40), a novel potential inflammatory biomarker, has been found to be elevated in very mild AD (94-96). In AD, there are also alterations in lipid metabolism. In spite of technically demanding detection approaches, biomarkers like F2-isoprostanes, 27-hydroxycholestrol (27OHC), ApoE, ApoJ, ApoA-I, and sphingolipids could serve as reliable biomarkers of AD (97-100).

Simultaneous measurement of many AD biomarkers and the search for novel biomarkers could be facilitated by protein profiling of the CSF. Nowadays, this has been enabled by advanced proteomics techniques like 2D gel electrophoresis, protein microarrays, immunoprecipitation, various types of mass spectrometry (surface-enhanced laser desorption/ionization-time of flight mass spectrometry [SELDI-TOF MS], matrix-assisted laser desorption/ionization-time of flight mass spectrometry [MALDI-TOF MS], liquid chromatography-mass spectrometry [LC-MS]), and stable isotope labeling kinetics (SILK) (95,101-105). In fact, using 2D-DIGE (2D-difference gel electrophoresis) and LC-MS/MS techniques, Perrin et al (96) detected 47 new potential AD biomarkers in the CSF, 4 of which were additionally confirmed by ELISA. Using LC-MS, Ringman et al (106) detected 56 proteins with altered expression in the CSF of AD patients (46 increased and 10 decreased). In addition, Simonsen et al (107) using MS detected a panel of 17 proteins and peptides for differentiation between patients with stable MCI and MCI-AD.

### CSF neurotransmitters in AD

Various studies have demonstrated the presence of perturbed neurotransmitter pathways in AD (108,109). As progressive failure of neuronal networks and neurotransmitter systems is one of the prominent features of AD, it is not surprising that in the CSF of AD patients abnormal concentrations of neurotransmitters and their metabolites have been found. Hence, many studies tried to assess the diagnostic potential of CSF neurotransmitters (110,111). However, the results on CSF monoamine metabolites in AD patients are conflicting (112,113). Moreover, despite the decreased brain noradrenaline content and loss of noradrenergic neurons in the locus coeruleus detected in AD, CSF markers of noradrenergic metabolism have not been proven diagnostically useful (111,114). Conflicting results have also been obtained on CSF γ-aminobutyric acid (GABA), glutamate, and neuropeptides (110,115,116). Although previous studies demonstrated inconsistent findings, the implementation of new methods for determination of different neurotransmitters and their metabolites in the CSF, such as LC-MS, showed some promising results (107,110,111). Namely, using this sensitive method, significant changes in the CSF levels of two important neurotransmitters/metabolites, adrenaline and 5-hydroxyindoleacetic acid (5-HIAA), which correlated with degeneration progression, were detected in a rat model for human tauopathy (117). Our study stressed the importance of early noncognitive, behavioral, and psychological symptoms of dementia that are caused by perturbed function of the brainstem (118). More precisely, it is considered that many behavioral and psychological symptoms of dementia (confusion, depression, agitation, disturbances in mood, appetite, emotion, and wake-sleep cycle) are caused by early degeneration of serotonergic raphe nuclei (118).

As selective loss or impairment of cholinergic neurons represents an important aspect of AD, the CSF markers of cholinergic activity have been extensively investigated. Even though previous studies on CSF cholinergic markers obtained conflicting data (119,120), recent reports have demonstrated alterations in the molecular forms and glycosylation patterns of acetylcholinesterase (AChE) in the CSF of AD patients, which reflect changes in the brain and might be useful as a marker of AD progression (121). It has been hypothesized that different AChE species and variants differ in their responses to disease and their interactions with Aβ and abnormally hyperphosphorylated tau. Namely, accumulating evidence suggests that both Aβ and p-tau can trigger an AChE increase in the regions around amyloid plaques and NFTs, which can in turn influence presenilin 1 (PSEN1) and thereby modulate Aβ production (122). Moreover, according to some authors, low butyrylcholinesterase (BuChE) levels found in AD patient's CSF are inversely related to BuChE in cortical amyloid plaques and could possibly predict extensive incorporation in neuritic plaques, increased neurotoxicity, and greater central degeneration (123). High ApoE and low BuChE levels in CSF strongly correlate with decreased cerebral metabolic rate of glucose consumption (CMRglc), high cerebral Aβ load, and CSF p-tau of patients with probable AD. These findings indicate that abnormally high levels of ApoE might play a causative role in the pathological events of AD, particularly those involving the early cholinergic deficit in the AD brain, through modulation of cholinesterases activities, hence disturbing the acetylcholine-dependent activity of neurons and glial cells (124).

Because of the methodological limitations and differences between studies, CSF neurotransmitters did not have high enough specificity and sensitivity to be considered as favorable biomarkers for AD. The observed discrepancy between the results obtained in various studies on CSF neurotransmitters and their metabolites or enzymes involved in synthesis or degradation in AD could partially be explained by the impact of the post-mortem changes, the origin of the CSF samples (ventricular vs lumbar post-mortem CSF), or the different determination methods used. Although no individual CSF neurotransmitter changes were found to be specific for AD, it may be possible to develop a profile of several neurochemical parameters (111) with enhanced sensitivity and specificity, which could improve AD diagnosis with currently established biomarkers. Broader biomarker investigations should lead to a better understanding of early disease mechanisms and the diagnosis of AD in the preclinical stages (125,126). Additionally, it should not be ignored that distribution of various substances along the CSF spaces depends on the rate of their removal into microvessels: faster removal means more limited distribution (127). Several studies demonstrated that organic acids pass freely between central nervous system (CNS) and CSF and vice versa and that active transport across capillary walls acts as a “sink” in their elimination from CNS and CSF. Thus, organic acids (for example 5-HIAA) pass from cisternal CSF into the CNS parenchyma, where they are being eliminated into capillaries by means of a powerful active transport, resulting in a swift decrease of their concentration inside the cisternal CSF, so they cannot be significantly distributed to remote CSF compartments (lumbar subarachnoid space) (128-131). This observation suggests that lumbar CSF concentration of neurotransmitter's metabolites better reflects local changes inside the spinal cord tissue than metabolic activity of upper CNS compartments.

## Other biomarkers of AD

### Neuroimaging biomarkers

In addition to CSF biomarkers, the most promising biomarkers of AD proved to be neuroimaging biomarkers (14,132). Magnetic resonance imaging (MRI) is a structural imaging technique that reveals abnormalities in the brain structure in high resolution. The earliest change in AD brain that can be detected using MRI is atrophy of the hippocampus and entorhinal cortex. But since these changes have also been detected in FTD and VaD, only MRI is not sufficient for the diagnosis of AD (133-135). Functional MRI (fMRI) used for the measuring alterations in the brain blood flow occurring due to neuronal activity has been recently considered as a method in diagnostics of dementia. AD patients had decreased neuronal activity in the hippocampus and parietal lobe, while neuronal activity in the primary (idiotypic) cortex, unaffected in AD, was increased (136). Although fMRI detected alterations in functional connectivity of the fusiform gyrus to the areas within the ventral and dorsal visual pathways in MCI patients, this method is still not applicable in diagnostics of dementia due to high inter- and intra-individual variability (137). Our recent study (138) has stressed the importance of fMRI in default mode network (DMN) imaging as a possible early new biomarker of AD. DMN, a major resting-state network in our brain is innervated by long projection fibers of noradrenergic, serotonergic, and cholinergic neurons from the brain stem that can release high amounts of Aβ in DMN hub regions. Additionally, due to their constant activity, neurons from DMN regions produce and release more Aβ than they do elsewhere in the cortex. This Aβ overload can lead to a functional impairment of DMN, which can be detected by fMRI very early, before the first dementia symptoms occur (138).

Of all neuroimaging techniques, the most promising is positron emission tomography (PET), which measures alterations in brain metabolism. Using this technique hypometabolism was reported, namely reduced cortical FDG (^18^F-fluorodeoxyglucose) uptake in the parietal, temporal, and posterior cingulate cortex of AD patients (139). Also, this method reached specificity and sensitivity of 93% and 84%, respectively, in differentiating AD from healthy control patients (140). Using other PET radiotracers – PiB (Pittsburgh Compound-B) and [^18^F]FDDNP (2-(1-{6-[(2-[^18^F]fluoroethyl)(methyl) amino]-2-naphthyl}ethylidene)malononitrile), this method can detect either senile plaques alone or both NFTs and senile plaques, respectively (141-144). Leuzy et al (145) stressed the possibility of amyloid PET imaging usage in personalized medicine. When combined with MRI, amyloid imaging showed very high specificity and sensitivity in early detection of AD. Also in addition to PiB and [^18^F]FDDNP, two more radiotracers have been approved for amyloid imaging: [^18^F]florbetapir (Amyvid) and [^18^F]flutemetamol (Vizamyl), plus other two still in phase III of clinical trials: [^18^F]-labeled florbetaben and [^18^F]-NAV4694 (138,145). Tau deposits have been visualized by [^18^F]-labeled T808 and [^11^C]-labeled phenyl/pyridinyl-butadienyl-benzothiazoles/benzothiazolium [PBB3] ligands that also reached phase III of clinical trials (138). The clinical application of selective tau imaging biomarkers is expected to become more and more important as it provides important information regarding tau pathophysiology in AD and non-AD tauopathies, allowing correlation of brain tau load with cognitive function, monitoring disease progression and evaluation of therapeutic efficacy of newly developed drugs aimed at modulating tau pathology (146).

Although neuroimaging methods reported excellent results in early detection and differentiation of AD, they are unfortunately still unavailable as a diagnostic tool in many clinical centers and hospitals due to the high costs of the technology itself and of the radiotracers. A significantly cheaper, but useful neuroimaging method – SPECT (single photon emission computerized tomography), commonly used for blood flow measurement, reliably revealed hypometabolism of the temporo-parietal and prefrontal cortices in AD patients in comparison to healthy elderly controls (147). Unfortunately, in spite of its wide availability, SPECT is still underused in the assessment of AD and related dementias.

### Plasma biomarkers

Numerous studies searched for reliable AD biomarkers in blood (plasma rather than serum), because lumbar puncture is still considered a relatively invasive method. Unfortunately, none of the potential plasma biomarkers is prognostically or diagnostically adequate due to their bioavailability. Those biomarkers that finally enter the plasma are highly diluted and adhere to various proteins (148). Aβ is the most studied plasma biomarker of AD. However, different studies yielded conflicting results, to the extent that some studies observed an increase in plasma Aβ_1-42_ and Aβ_1-40_, increase in Aβ_1-42_ (but not Aβ_1-40_), increase in Aβ_1-40_ (but not Aβ_1-42_), or unaltered levels of both proteins (148,149). Decreased Aβ_1-42_ /Aβ_1-40_ ratio was also reported as a risk factor for MCI conversion to AD (150). In fact it remains unclear whether plasma Aβ truly reflects the situation in the brain because Aβ is also produced elsewhere in the body (151).

Plasma levels of α2-macroglobulin, complement factor H, homocysteine, cholesterol, F2-isoprostanes, Aβ autoantibodies, and ApoA1 have also been measured (148,152-155). None of these potential biomarkers reached satisfying sensitivity and specificity. However, Manzine et al (156) observed reduced expression of platelet ADAM10 (A Disintegrin and Metalloprotease), stressing the possibility of its use as an early biomarker of AD. It is believed that a combination of plasma biomarkers could result in diagnostically useful screening tests. Thus, only patients with suspected dementia would be subjected to determination of highly specific and sensitive neuroimaging and CSF biomarkers (157).

### Genetic biomarkers

Familial AD (prevalence around 0.1%) is related to mutations in the genes for APP, *PSEN1*, and presenilin 2 (*PSEN2*) (158). However, the genetic causes of sporadic AD are not yet understood (159). The only well-known risk factor for AD is the ϵ4 allele of *APOE* gene. One ϵ4 allele triples the risk of AD, while two ϵ4 alleles increase the risk 15 times (160). Using genome-wide association studies (GWAS), scientists have attempted to detect new gene variants involved in the emergence of sporadic AD (161,162). In fact, a recent study detected 120 gene loci associated with AD (163). Cruchaga et al (164) reported that the presence of a rare variant Val232Met in *PLD3* (phospholipase D3) doubles the risk for sporadic AD. As PLD3 influences APP processing, any impairment of PLD3 function leads to the aberrant APP processing.

The other approach is comparison of gene expression between AD patients and healthy controls. Gene expression profiling can be done by using the RNA extracted from the cells precipitated in the pellets of CSF samples or peripheral blood. As a large number of studies reported altered gene expression in AD, this approach is also considered for AD diagnosis (165,166).

## Combination of biomarkers

While measurement of single biomarkers (especially p-tau) resulted in very high sensitivity and specificity, combining more CSF biomarkers can improve diagnostic accuracy (Table 3) (8,23,38,95,141,167-173). Shaw et al (171) combined Aβ_1-42_, t-tau, and p-tau_181_ with the purpose of establishing a “signature of AD,” resulting in mixed data. The Luminex xMAP technology (Luminex, Austin, TX, USA) used in the measurement of Aβ_1-42_, t-tau, and p-tau_181_ in increasingly more studies proved as accurate as standard ELISA methods. The additional and preferable characteristic of this technology is that it measures all CSF biomarkers in the same CSF aliquot (174). Spies et al (175) developed a very accurate prediction model for determination of AD probability among individuals suspected of dementia. This model, based on logistic regression analysis, calculates the probability of AD from the levels of CSF biomarkers (Aβ_1-42_ and p-tau_181_) and the patients’ gender. Variability in the values of CSF biomarkers due to differences in pre-analytical and analytical procedures or differences in ELISA kits from various manufacturers still represents a major problem. While analytical factors refer to laboratory procedures in different laboratories (176), pre-analytical variability is related to the selection of participants and treatment of CSF samples after lumbar puncture (eg, the tube type, storage temperature, and the number of freeze/thaw cycles before analysis) (50,177,178). It is difficult to influence the variability caused by differences among ELISA kits of various manufacturers (179). Our recent study indicates that there are differences between t-tau and Aβ_1-42_ ELISA kits from different vendors, making it impossible to use them interchangeably (26). Fagan et al (143) also reported differences in the absolute values of CSF Aβ_1-42_, t-tau, and p-tau_181_ after measurement by the two most frequently used methods (INNOTEST ELISA and INNO-BIA AlzBio3 assay on Luminex xMAP technology [Luminex]). The Alzheimer Association in 2009 initiated an international quality control program for CSF biomarkers. The results for 2010-2012 showed that coefficients of variation among laboratories are still very high, ranging from 20% to 30% (180). Thus, the debate is still on if the established cut-off levels for Aβ_1-42_, t-tau, and p-tau_181_ should be widely used (32) or every laboratory should define internally qualified cut-off levels (180).

**Table 3:** Characteristics of biomarker combinations used for diagnosing Alzheimer disease

Combination of biomarkers		Observations	References
**CSF**	Aβ_1-42_ t-tau	Sensitivity 85%-94%, specificity 83%-100% in differentiating AD from HC	(8)
	t-tau/Aβ_1-42_ ratio	1. Sensitivity 89% in detection of MCI-AD patients 2. More accurate prediction of conversion from normal or MCI to AD	1 (171). 2 (95,141,169).
	p-tau_181_/Aβ_1-42_	1. Differentiation of AD from HC – sensitivity 86%, specificity 97%; Differentiation of AD from other dementias – sensitivity 80%, specificity 73% 2. Better prediction of MCI conversion to AD	1 (168). 2 (141).
	Aβ_1-42_ t-tau p-tau_181_	1. Sensitivity 95%, specificity 83% in detection of MCI-AD patients 2. Sensitivity 83%, specificity 72% for differentiation of MCI-AD patients from stable MCI 3. Luminex xMAP technology for simultaneous measurement of all 3 core CSF biomarkers	1 (38). 2 (170). 3 (174,184).
	p-tau_181_ Aβ_1-42_/Aβ_1-38_ ratio	Sensitivity 94%, 85% specificity in differentiating AD from other primary causes of dementia	(172)
	Aβ_1-42_, t-tau F2-isoprostanes	Sensitivity 84%, specificity of 89% in differentiating AD from HC and other dementias	(167)
**CSF + neuroimaging**	Aβ_1-42_ PiB-PET	Decreased CSF Aβ_1-42_ and increased PiB binding in the brain of AD patients	(27,142,185)
	t-tau/Aβ_1-42_, p-tau/Aβ_1-42_ PiB-PET	Better correlation of these ratios than Aβ_1-42_ alone with PiB binding in the brain of AD patients	(143,186)
	Aβ_1-42_ t-tau, p-tau_181_ MRI	Association of: 1. WBA with decrease of Aβ_1-42_ in preclinical AD plus correlation of t-tau and p-tau_181_ with further atrophy caused by disease progression 2. Hippocampal volume with CSF t-tau and p-tau	1 (24) 2 (187)
	t-tau/p-tau_181_ Aβ_1-42_ MRI	Results of longitudinal study indicated that higher normal WBA slows the occurrence of dementia symptoms in individuals with pathological values of CSF biomarkers	(188)
	t-tau/p-tau_181_ Aβ_1-42_ rCBF	MCI patients with reduced regional cerebral blood flow in the parietal cortex and pathological levels of CSF biomarkers had higher risk of AD	(189)
	t-tau, p-tau_231_ isoprostanes Aβ_1-42_/Aβ_1-40_ MRI	More accurate detection of MCI-AD patients by combination of CSF biomarkers and measurement of medial temporal lobe atrophy using MRI. 74%-84% accuracy of MCI-AD detection after combination of p-tau_231_ and MRI.	(190)
	VILIP-1 t-tau, p-tau_181_ Aβ_1-42_ MRI, PiB-PET	CSF VILIP-1 positively correlated with tau, p-tau_181_ and PiB binding and negatively with WBA. VILIP-1/Aβ_1-42_ predicted cognitive impairment as well as p-tau_181_/Aβ_1-42_ and tau/Aβ_1-42_	(173)
**CSF + neuroimaging + genetic testing**	Aβ_1-42_ PIB-PET *APOE* genotype	*APOE* ϵ*4* allele carriers had elevated PiB binding in the brain and decreased CSF Aβ_1-42_, while *APOE* ϵ*2* allele had protective effect	(191)
	PIB-PET *APOE* genotype	Increased PiB binding in the brain of *APOE* ϵ*4* allele carriers	(192,193)
	BACE1 activity *APOE* genotype	Increased BACE1 activity at *APOE* ϵ*4* allele carriers	(194)
	Aβ_1-42_, p-tau_181_, *BIN1, CLU*, *CR1*, *PICALM* genotype	Variants of *BIN1, CLU, CR1* and *PICALM* genes associated with susceptibility for AD do not affect CSF Aβ_1-42_ and p-tau_181_	(195)
	p-tau_181_, *PPP3R1* genotype	Rs1868402 variant of *PPP3R1* gene associated with higher levels of p-tau_181_ and faster progression of AD	(196)

*Abbreviations: CSF – cerebrospinal fluid; Aβ – amyloid β; AD – Alzheimer disease; HC – healthy controls; MCI – mild cognitive impairment; PiB – Pittsburgh Compound-B; WBA – whole brain atrophy.

Detection of AD in asymptomatic individuals is still very difficult, even in specialized centers. With the emergence of new drugs for AD, it is likely that the diagnosis will be based on combinations of different biomarkers (36). Dubois et al (16) suggested that for diagnosing AD, *APOE* genotype and neuroimaging biomarkers should be determined besides CSF biomarkers. Therefore, recent efforts on AD biomarkers have focused on improving AD diagnosis by a combination of different biomarkers (CSF, neuroimaging, and genetic biomarkers) (Table 3).

A recent MEDLINE search for the most common biomarkers of AD (Aβ, tau, MRI, PIB-PET, FDG-PET) performed by Noel-Storr et al (181) resulted in 19 104 published references, 1032 of which were cross-sectional studies, 500 longitudinal studies, while the rest of the publications was not relevant. Because of the number of studies performed on AD biomarkers, scientists also use meta-analysis as a tool for the assessment of biomarker variability or validity and to stress the need for methodology standardization among investigations, with an ultimate goal to facilitate the difficult process of biomarker validation (37,181,182).

## Conclusions

Determination of different biomarkers for AD is expensive and unfortunately still untenable in many institutions. Due to a non-invasive method of sample collection, the best choice are considered to be biomarkers measured in blood (plasma or serum) or urine. Unfortunately, these biomarkers showed little accuracy in diagnostics of AD (183). The second choice are CSF biomarkers because lumbar puncture is still considered as an invasive procedure. Neuroimaging biomarkers are the last choice due to the usage of expensive radiotracers and sophisticated techniques that are still not widely available. For comparison, MRI and PIB-PET are 3 to 25 times more expensive, respectively, than measurements of Aβ_1-42_, t-tau, and p-tau_181_ concentration in the CSF (Table 1). However, the diagnostic potential of these well-established core CSF biomarkers should be further improved by novel CSF biomarkers, warranting further studies on their detection and evaluation. This should decrease the age limit of AD detection, enable disease detection in preclinical stage (Figure 1), and consequently facilitate the administration of potential therapeutics to AD patients before irreversible degeneration occurs.
